# Supplementary material for: Optogenetic induction of mechanical muscle stress identifies myosin regulatory ubiquitin ligase NHL-1 in C. elegans
Source: Nat Commun. 2024 Aug 11;15:6879. doi: 10.1038/s41467-024-51069-3 (PMC11317515; doi:10.1038/s41467-024-51069-3)
Supplement: Supplementary file 1 — Supplementary Information [file 41467_2024_51069_MOESM1_ESM.pdf]

Optogenetic induction of mechanical muscle stress identifies myosin regulatory ubiquitin ligase NHL-1 in *C. elegans*

Carl Elias Kutzner[1,2,3], Karen Carolyn Bauer[1,2], Jan-Wilm Lackmann[2], Richard James Acton[4], Anwesha Sarkar[5], Wojciech Pokrzywa[5], Thorsten Hoppe[1,2,3,*]

[1] Institute for Genetics, University of Cologne, Cologne, Germany

[2] Cologne Excellence Cluster for Cellular Stress Responses in Aging-Associated Diseases (CECAD), University of Cologne, Cologne, Germany

[3] Center for Molecular Medicine Cologne (CMMC), University of Cologne, Cologne, Germany

[4] Human Developmental Biology Initiative (HDBI) at Babraham Institute, Cambridge, United Kingdom

[5] Laboratory of Protein Metabolism, International Institute of Molecular and Cell Biology in Warsaw, Warsaw, Poland

*Corresponding author. Email: thorsten.hoppe@uni-koeln.de

Supplementary Files include

Supplementary Figures S1 to S7

Supplementary Movies V1 and V2 as separate MP4 files

Supplementary Tables 1 to 9 as one separate Supplementary Data Microsoft Excel file

Supplementary Methods

Supplementary References

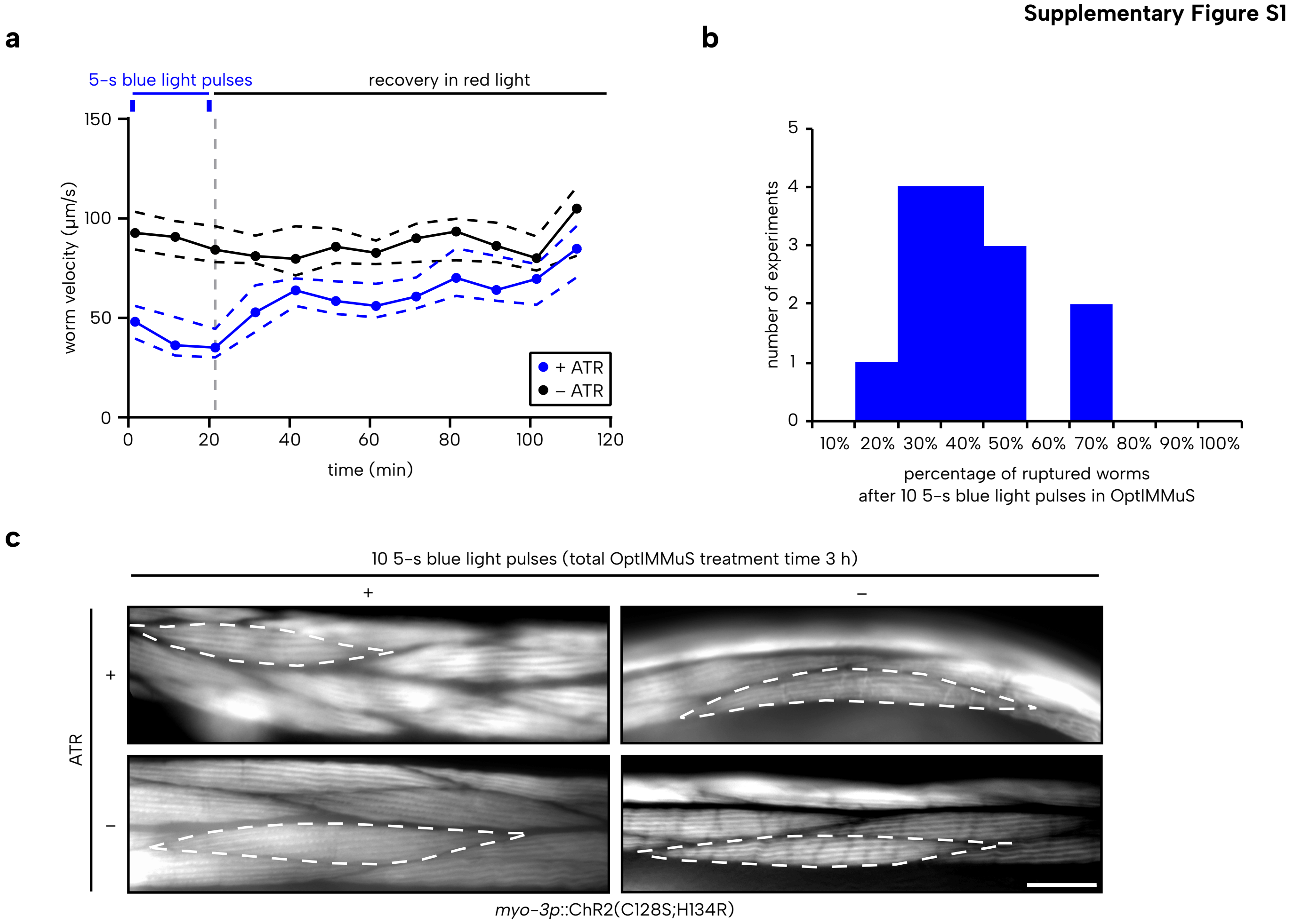
a
5-s blue light pulses
recovery in red light
150
100
50
0
worm velocity (µm/s)
0
20
40
60
80
100
120
time (min)
+ ATR
– ATR
b
5
4
3
2
1
0
number of experiments
10% 20% 30% 40% 50% 60% 70% 80% 90% 100%
percentage of ruptured worms
after 10 5-s blue light pulses in OptIMMuS
c
10 5-s blue light pulses (total OptIMMuS treatment time 3 h)
+
–
ATR
+
–
myo-3p::ChR2(C128S;H134R)

Supplementary Figure S1. OptIMMuS triggers sustained muscle contraction and vulval rupture in *C. elegans*. a, Worm velocity in µm per second measured in the WormLab system from 75 individual worms showing reduced movement after two consecutive 5-s blue light pulses interrupted by a 20-min red light phase and slow recovery in the hour following the second pulse. Values are median (n = 75) with 95% CI as interrupted lines. **b**, Histogram showing the frequency of occurrence of the percentage of worms with vulval rupture in 14 independent OptIMMuS experiments. **c**, Fluorescence micrographs of rhodamine-phalloidin-stained body wall muscle (BWM) cells in worms after 10 5-s blue light pulses interrupted by 20-min dark recovery phases on ATR and control worms without ATR or without blue light exposure. A single BWM cell is outlined with an interrupted white line. Scale bar: 20 µm. Source data are provided as Source Data file.

Supplementary Figure S2

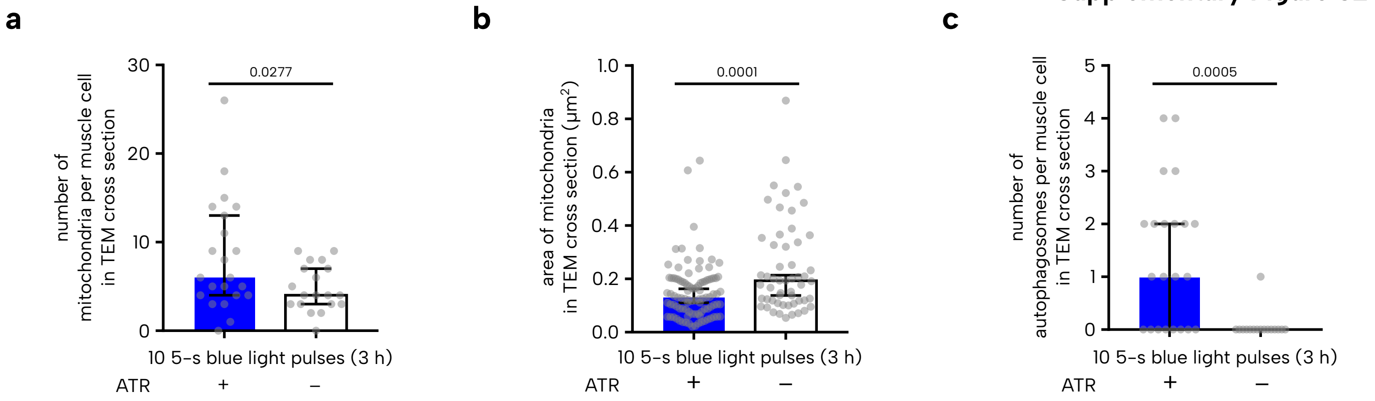

Supplementary Figure S2. OptIMMuS-triggered sustained contractions stress muscle cells, alter mitochondrial morphology, and increase the number of autophagosomes. a, Bar graphs showing increased numbers of mitochondria per body wall muscle (BWM) cell in transverse TEM sections in worms treated with ATR and 10 5-s blue light pulses interrupted compared to control without ATR. Values are median (*n* = 22 and 19 cells [left to right] imaged in *n* = 2 individual worms per condition) with 95% CI. **b**, Bar graphs showing decreased areas of mitochondria per BWM cell in transverse TEM sections in worms treated with ATR and 10 5-s blue light pulses interrupted compared to control without ATR. Values are median (*n* = 96 and 54 mitochondria [left to right] in *n* = 11 and 10 cells, respectively, imaged in *n* = 2 individual worms per condition) with 95% CI. **c**, Bar graphs showing increased numbers of autophagosomes per BWM cell in transverse TEM sections in worms treated with ATR and 10 5-s blue light pulses interrupted compared to control without ATR. Values are median (*n* = 22 and 16 cells [left to right] imaged in *n* = 2 individual worms per condition) with 95% CI. In **a**, **b** and **c**, p-values compared to control in two-tailed unpaired Student's t-tests. Source data are provided as Source Data file.

Supplementary Figure S3

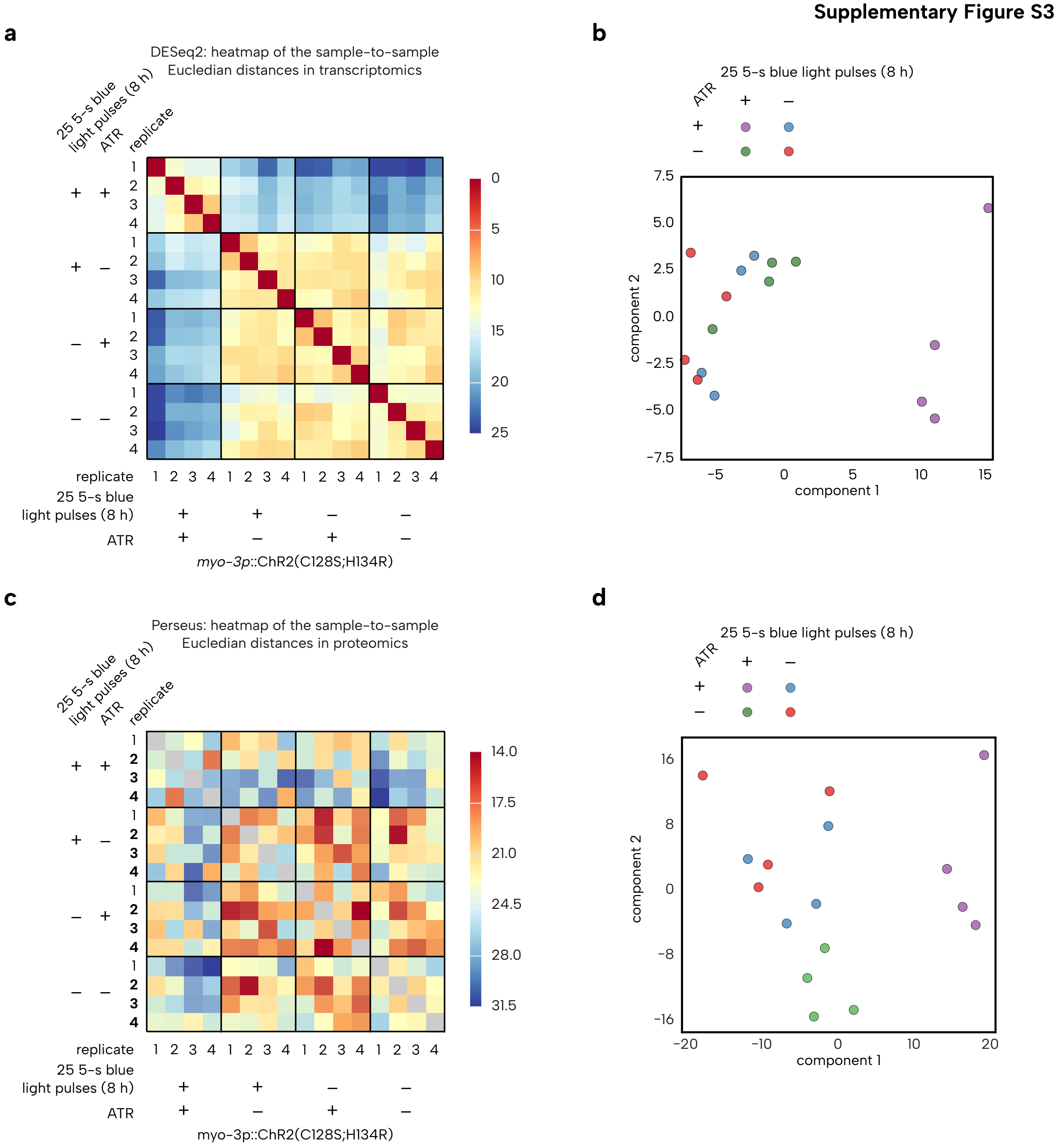

Supplementary Figure S3. OptIMMuS-triggered sustained contractions rewire the transcriptome and proteome, resulting in distinct molecular changes compared to the three controls in an OptIMMuS experiment. **a**, Heatmap of sample-to-sample Eucledian distances in transcriptomics showing the similarity of the three controls in the OptIMMuS experiment. Calculated in DEseq2 (v1.30.1)[1] and generated with MultiQC. **b**, Principal component analysis of transcriptomics samples. Calculated in DEseq2 (v1.30.1)[1] and generated with MultiQC. **c**, Heatmap of sample-to-sample Eucledian distances in proteomics showing the similarity of the three controls in the OptIMMuS experiment. Calculated in Perseus 1.6.15[2]. **d**, Principal component analysis of proteomics samples. Calculated in Perseus 1.6.15[2] and visualized in InstantClue[3]. Source data are provided as Source Data file.

Supplementary Figure S4

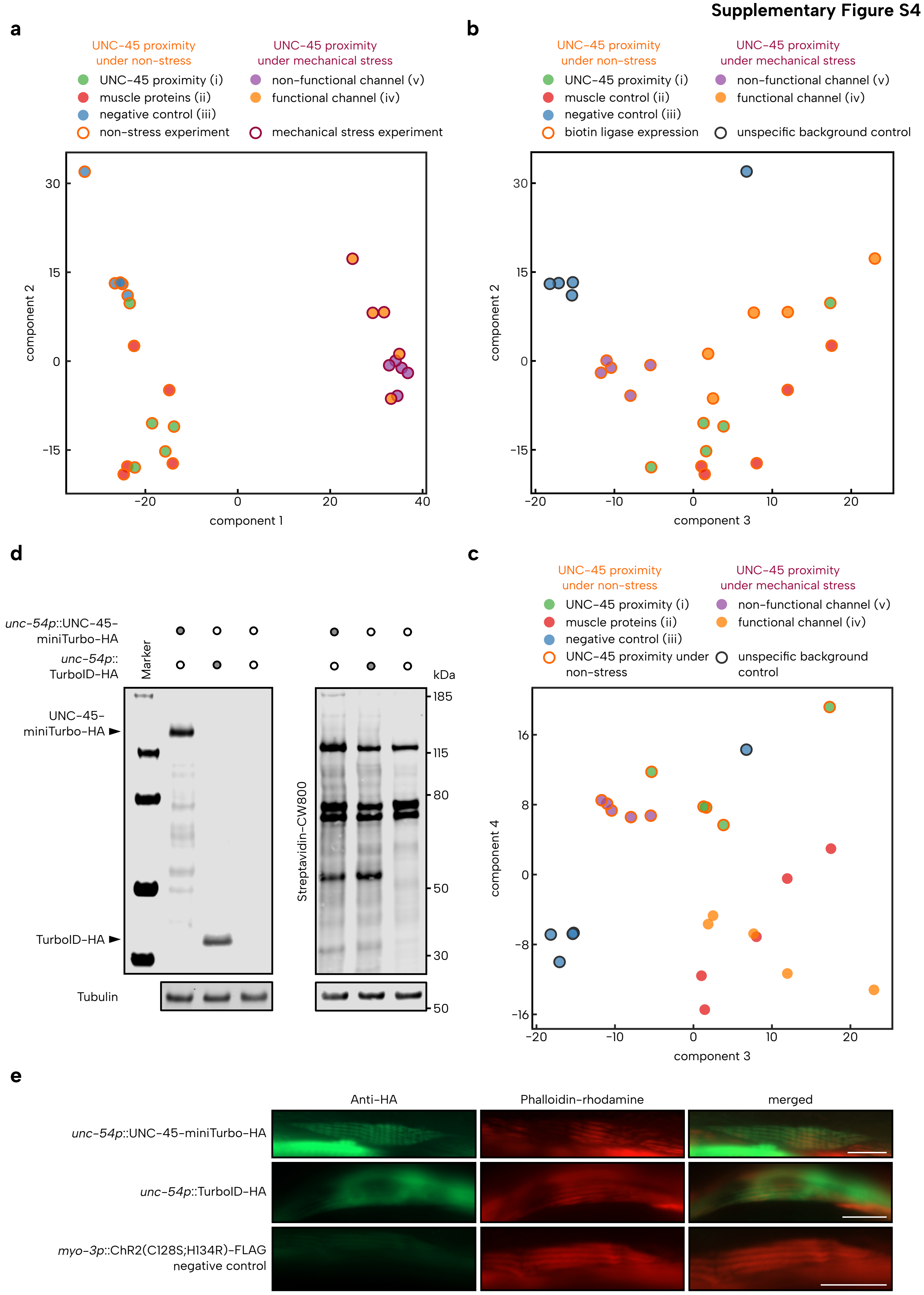

Supplementary Figure S4. Principal component analysis of proximity proteomics samples allows the combination of the mass spectrometry experiments in subsequent analyses. Principal component analysis was calculated in Perseus 1.6.15[2] and visualized in InstantClue[3]. **a**, Principal component 1 separates the samples according to non-stress and mechanical stress experiments. **b**, Principal components 2 and 3 separate the samples according to biotin ligase expression or background control of endogenously biotinylated proteins and unspecific binders. **c**, Principal components 3 and 4 show the similar clustering of the UNC-45 proximity samples under non-stress conditions in both experiments. **d**, Western blot of muscle-expressed UNC-45-miniTurbo-HA, TurboID-HA, biotinylated proteins, and the housekeeping protein tubulin in lysates of transgenic worms shows similar expression levels of the biotin ligases and comparable biotinylation patterns enhanced over endogenous background biotinylation in the control without biotin ligase expression. Representative result of n = 3 independent experiments. **e**, Fluorescence micrographs of Anti-HA and rhodamine-phalloidin-stained body wall muscle cells in biotin ligase-expressing worms shows expression of UNC-45-miniTurbo and TurboID in muscle cells. Scale bars: 20 µm. Representative result of n = 1 independent experiments. Source data are provided as Source Data files.

a

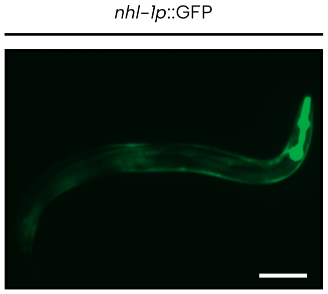
nhl-1p::GFP

b

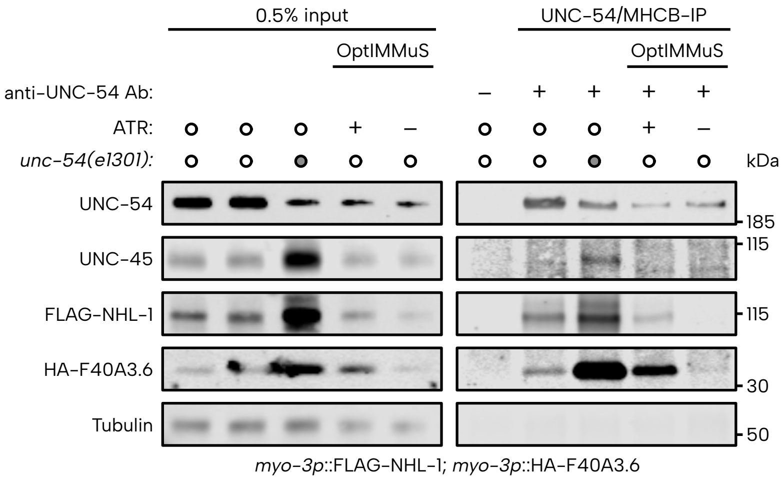
0.5% input
UNC-54/MHCB-IP
OptIMMuS
OptIMMuS
anti-UNC-54 Ab:
ATR:
unc-54(e1301):
kDa
UNC-54
UNC-45
FLAG-NHL-1
HA-F40A3.6
Tubulin
185
115
115
30
50
myo-3p::FLAG-NHL-1; myo-3p::HA-F40A3.6

c

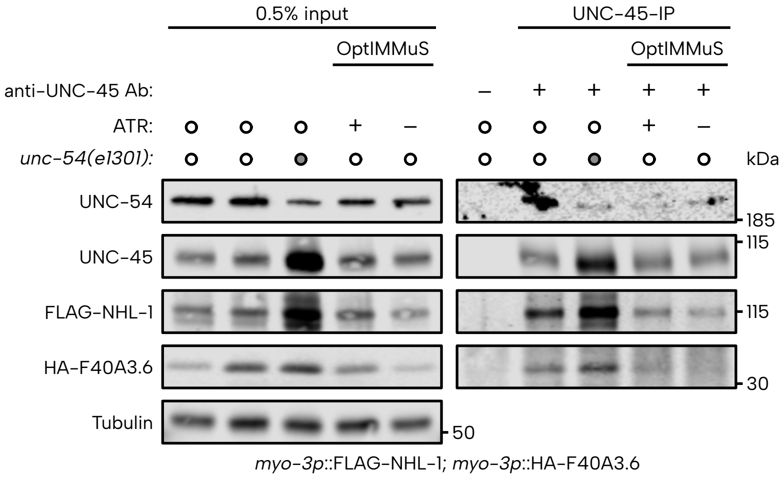
0.5% input
UNC-45-IP
OptIMMuS
OptIMMuS
anti-UNC-45 Ab:
ATR:
unc-54(e1301):
kDa
UNC-54
UNC-45
FLAG-NHL-1
HA-F40A3.6
Tubulin
185
115
115
30
50
myo-3p::FLAG-NHL-1; myo-3p::HA-F40A3.6

Supplementary Figure S5. NHL-1 and F40A3.6 physically interact with UNC-45 and muscle myosin. **a**, Fluorescence micrograph of a young adult worm carrying an extrachromosomal *nhl-1p*::GFP transcriptional reporter transgene shows *nhl-1* expression in body wall muscle cells and pharynx. Representative result of *n* = 2 independent experiments. Scale bar: 100 µm. **b**, Western blot after immunoprecipitation of myosin/UNC-54 in wild-type (WT) and *unc-54(e1301)* worms and after 13 5-s blue light pulses in OptIMMuS shows the binding of UNC-45 and transgenically muscle-expressed FLAG-NHL-1 and HA-F40A3.6. Representative result of *n* = 3 independent experiments. **c**, Western blot after immunoprecipitation of UNC-45 in WT and *unc-54(e1301)* worms and after 13 5-s blue light pulses in OptIMMuS shows the binding of UNC-54/myosin and transgenically muscle-expressed FLAG-NHL-1 and HA-F40A3.6. Representative result of *n* = 3 independent experiments. Source data are provided as Source Data file.

Supplementary Figure S6

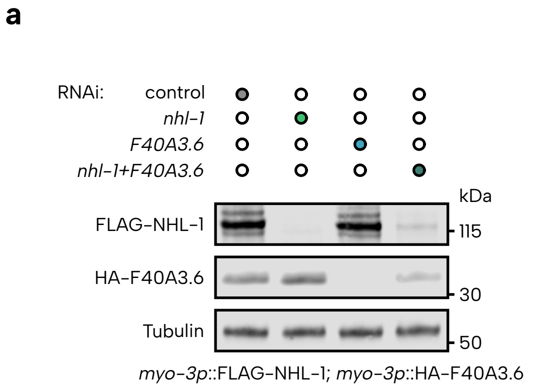

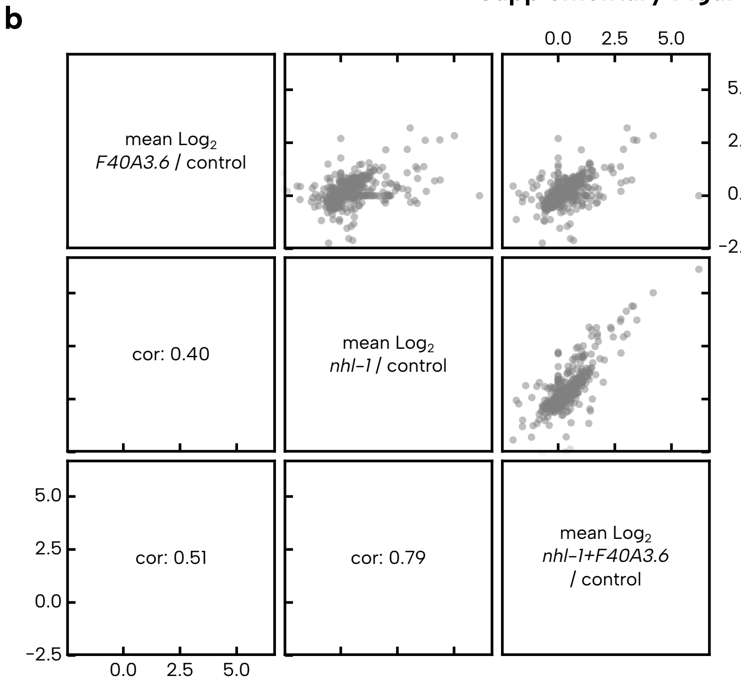

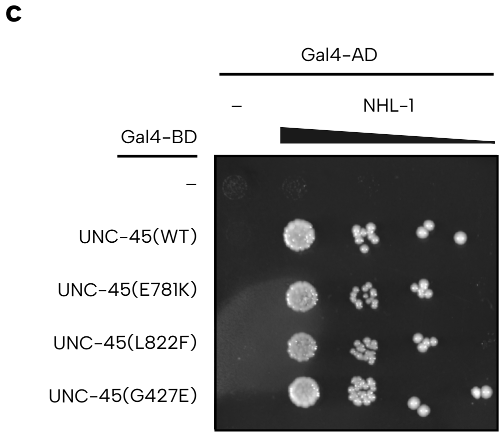

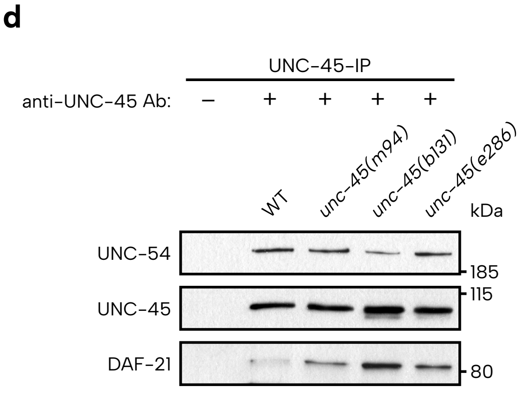

Supplementary Figure S6. Efficiency of *nhl-1* and *F40A3.6* RNAi treatments alone and together from the same plasmid and interaction of UNC-45 mutant proteins with NHL-1 and myosin. a, Western blot of muscle-expressed FLAG-NHL-1, HA-F40A3.6, and the housekeeping protein tubulin in lysates of transgenic worms shows a higher efficiency of the RNAi-mediated depletion of *nhl-1* and *F40A3.6* individually than together from the same plasmid compared to control. Result of n = 1 experiment is shown. **b**, RNAi-mediated changes in co-immunoprecipitated UNC-54 interactors show a wider spread upon *nhl-1* RNAi compared to control and a higher correlation between *nhl-1* and *nhl-1+F40A3.6* RNAi compared to control. Correlations calculated in InstantClue[3]. **c**, Yeast grown on an SD/–Leu/–Trp/–His yeast two-hybrid agar plate after 5 days at 30°C show growth and binding of NHL-1 to UNC-45 WT and mutant proteins. Representative result of n = 3 independent experiments. **d**, Western blot after immunoprecipitation of UNC-45 in wild-type (WT) and *unc-45* mutant worms shows reduced binding of UNC-54/myosin in *unc-45(b131)* worms. Representative result of n = 2 independent experiments. Source data are provided as Source Data file.

Supplementary Figure S7

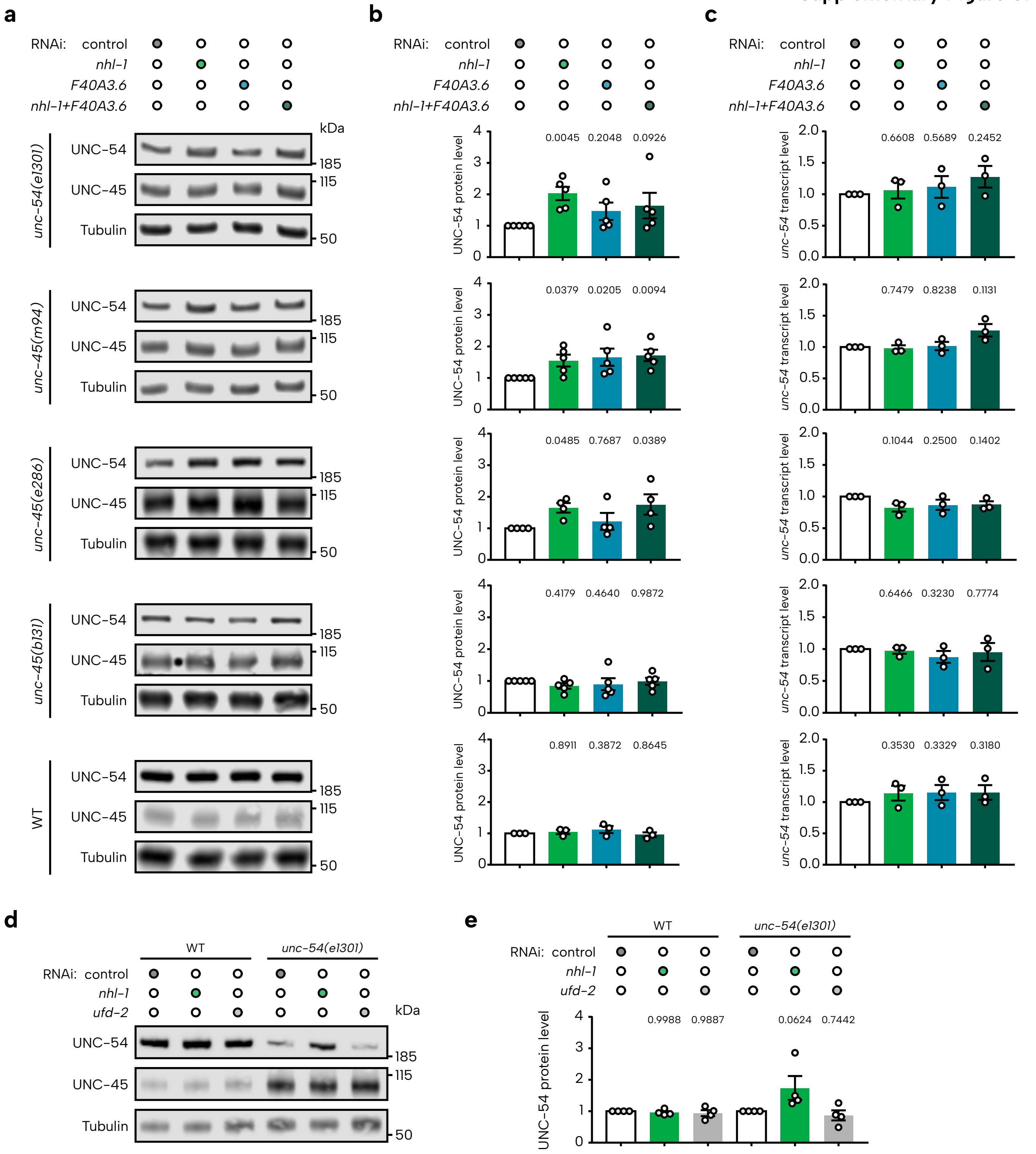

Supplementary Figure S7. The E3 ligase NHL-1 and its binding partner F40A3.6 regulate myosin protein levels. a, Western blots of UNC-54/myosin, UNC-45, and the housekeeping protein tubulin in lysates from myosin misfolding mutants show an increase in myosin/UNC-54 protein levels but not UNC-45 levels upon RNAi-mediated depletion of *nhl-1* compared to control. Representative results of *n* = 5, 5, 4, 4, 3 independent experiments (top to bottom). The Western blot for *unc-54(e1301)* from Fig. 5e is shown here again for completeness. **b**, Quantification of UNC-54/myosin protein levels detected as in **a** normalized to tubulin protein levels and to RNAi control. Values are mean (*n* = 5, 5, 4, 4, 3 independent experiments, top to bottom) with SEM; p-values compared to control in repeated-measures one-way ANOVA with Dunnett's multiple comparisons test of log2-transformed values. The results for *unc-54(e1301)* from Fig. 5f are shown here again for completeness. **c**, Relative *unc-54* transcript levels determined by qRT-PCR show no significant change in endogenous expression levels in myosin misfolding mutants upon RNAi-mediated depletion of *nhl-1*, *F40A3.6*, or both compared to control. Values are mean (*n* = 3 independent experiments) with SEM; p-values of one-sample t-test against 1. **d**, Western blot of UNC-54/myosin, UNC-45, and the housekeeping protein tubulin in lysates from wild-type (WT) and the *unc-54(e1301)* myosin misfolding mutant worms shows an increase in myosin/UNC-54 protein levels upon RNAi-mediated depletion of *nhl-1* but not of *ufd-2* compared to control. Representative result of *n* = 4 independent experiments. **e**, Quantification of UNC-54/myosin protein levels detected as in **d** normalized to tubulin protein levels and to RNAi control. Values are mean (*n* = 4 independent experiments) with SEM; p-values compared to control RNAi in repeated-measures one-way ANOVA with Sidak's multiple comparisons test of log2-transformed values. Source data are provided as Source Data files.

Supplementary Methods

Microscopy video of a blue light pulse

For supplementary microscopy videos, the 5-s blue light pulse was generated manually with a CoolLED pE-300ultra light source using the blue LED (450 nm) and a liquid light guide. The light intensity was matched to the light intensity of 10 µW per mm^2 inside the OptIMMuS incubator by parallel measurement using the ThorLabs Power and Energy Meter PM100D with a S120C Photodiode Power Sensor (400-1100 nm, 50 mW) at the wavelength setting 450 nm (range 1.9 mW).

Transmission electron microscopy

After 10 5-s blue light pulses interrupted by 20-min dark recovery phases on plates with or without ATR (3 h OptIMMuS treatment time), 5–10 *C. elegans* worms were picked from a plate and transferred into a membrane carrier (Leica #16707898) prefilled with 20% polyvinylpyrrolidone in PBS. Carriers were mounted in sample pods and frozen using the EmPACT2 (Leica). Samples were stored in liquid nitrogen until further processing and analysis by the CECAD imaging facility. Freeze substitution was performed using the AFS 2 (Leica) with a mixture of 0.1% tannic acid (Sigma), 0.5% glutaraldehyde (Science Services) and 2% ddH_2O in aceton (Sigma) at –90°C. After 96 h, the samples were washed three times with acetone at –90°C and incubated with 2% osmium tetroxide in acetone for 35 h. The temperature was increased 5°C per hour and held at –20°C for 16 h. The temperature was increased 6°C per hour to 4 °C and the samples were washed four times with acetone and incubated overnight in propylene oxide (Sigma). Samples were infiltrated with 50% Epon (Sigma) in propylene oxide for 5 h, 90% Epon in propylene oxide overnight, and twice pure Epon for 3 h. Individual *C. elegans* worms were removed from the membrane carrier, mounted in flat embedding molds, filled with Epon, and cured at 60°C for 48 h. The head or tail region was cut longitudinally or transversely into 70 nm ultrathin sections using an ultramicrotome (Leica Microsystems, UC6)

and a diamond knife from Diatome (Science Services) and stained with 1.5% uranyl acetate (Agar Scientific) for 15 min at 37°C and 3% Reynolds lead citrate solution prepared from lead (II) nitrate (Roth) and trisodium citrate dehydrate (Roth) for 2 min. The sections were imaged on a JEM-2100Plus transmission electron microscope (JEOL). The number of mitochondria per body wall muscle cell, the area of each body wall muscle cell, and the number of autophagosomes per body wall muscle cell were measured in the images of cross-sections.

RNA-seq analysis

RNA-seq library preparation and sequencing were performed by the Cologne Center for Genomics (CCG), Germany (https://ccg.uni-koeln.de/). RNA quality after RNA extraction was determined using the TapeStation system (Agilent) with TapeStation Analysis Software 3.2 (Agilent). RNA-seq library preparation with poly(A) enrichment was performed on 2 µg total RNA using the TruSeq Stranded mRNA Prep kit (Illumina) including ERCC spike-in. Libraries were sequenced with 30 million 100 bp paired-end reads on an NovaSeq 6000 (Illumina). RNA-seq analyses were performed by the CECAD bioinformatics facility using the nf-core[4] RNA-seq pipeline (v3.0). Transcripts were mapped to the *C. elegans* genome WBcel235 from Ensembl (v103). Differential expression analysis of blue light plus ATR versus blue light minus ATR was performed in R (v4.0.3) using DESeq2 (v1.30.1)[1]. Estimation of log-fold change shrinkage estimation was performed with apeglm (v1.12.0). p-values were adjusted for multiple testing using the Benjamini-Hochberg procedure.

Whole worm mass spectrometry analysis

Samples in 8 M urea/50 mM TEAB buffer for whole proteome analysis (four conditions in four biological replicates as described for RNA-seq) were reduced with 5 mM DTT, alkylated with 40 mM CAA, and digested with 0.5 µg Lys-C and 1 µg trypsin. After overnight incubation, digestion was stopped by adding formic acid to 1%, peptides were loaded onto equilibrated

SDB-RPS stage tips and stored at 4°C until mass spectrometry analysis. All samples were analysed by the CECAD proteomics facility on a Q Exactive Plus Orbitrap mass spectrometer that was coupled to an EASY nLC (both Thermo Scientific). Peptides were loaded onto an in-house packed analytical column (50 cm, 75 µm inner diameter, filled with 2.7 µm Poroshell EC120 C18, Agilent) with solvent A (0.1% formic acid in water). Peptides were chromatographically separated at a constant flow rate of 250 nL/min using the following gradient: initial 4% solvent B (0.1% formic acid in 80% acetonitrile), 4–6% within 1.0 min, 6–30% solvent B within 200.0 min, 30–50% solvent B within 28.0 min, 50–95% solvent B within 1.0 min, followed by washing and column equilibration. The mass spectrometer was operated in data-dependent acquisition mode. The MS1 survey scan was acquired from 300–1750 m/z at a resolution of 70,000. The top 10 most abundant peptides were isolated within a 1.8 Th window and subjected to HCD fragmentation at a normalized collision energy of 27%. The AGC target was set to 5e5 charges, allowing a maximum injection time of 55 ms. Product ions were detected in the Orbitrap at a resolution of 17,500. Precursors were dynamically excluded for 40.0 s.

Pull-down of biotinylated proteins and mass spectrometry analysis

To determine the cellular network of transient UNC-45 interactors under non-stress conditions, worms were grown to adulthood at 15°C (three conditions in five biological replicates). To detect changes in the UNC-45 interaction network upon mechanical stress, worms grown on plates with or without ATR were exposed to 13 5-s blue light pulses interrupted by 20-min dark recovery phases (two conditions in five biological replicates, total OptIMMuS treatment time 4 h). Worms were then collected and washed, and worm pellets were snap frozen and stored at –80°C until further processing. Protein from all five independent biological replicates per experiment was isolated together by sonication on ice in modified RIPA buffer (50 mM Tris pH 7.5, 150 mM NaCl, 1 mM EDTA, 1 mM EGTA, 1% Triton X-100) with cOmplete protease inhibitor cocktail (Roche). Protein concentration in cleared lysates was measured using a Pierce

BCA protein assay kit (Thermo Fisher Scientific). 3 mg total protein was diluted in PBS and incubated with equilibrated Streptavidin Sepharose High Performance Beads (GE Healthcare) overnight at 4°C. The beads were washed five times in PBS, and on-bead Lys-C/trypsin digestion was performed in 8 M urea/50 mM TEAB buffer with cOmplete protease inhibitor cocktail (Roche) as for whole worm mass spectrometry, followed by mass spectrometric analysis. All samples were analysed by the CECAD proteomics facility on a Q Exactive Plus Orbitrap mass spectrometer that was coupled to an EASY nLC (both Thermo Scientific). Peptides were loaded onto an in-house packed analytical column (40 cm length, 75 µm inner diameter, filled with 2.7 µm Poroshell EC120 C18, Agilent) with solvent A (0.1% formic acid in water). Peptides were chromatographically separated at a constant flow rate of 250 nL/min using the following gradient: initial 3% solvent B (0.1% formic acid in 80% acetonitrile), 3–5% B within 1.0 min, 5–30% solvent B within 65.0 min, 30–50% solvent B within 13.0 min, 50–95% solvent B within 1.0 min, followed by washing and column equilibration. The mass spectrometer was operated in data-dependent acquisition mode. The MS1 survey scan was acquired from 300–1750 m/z at a resolution of 70,000 and a maximum injection time of 20 ms. The top 10 most abundant peptides were isolated within a 1.8 Th window and subjected to HCD fragmentation at a normalized collision energy of 27%. The AGC target was set to 5e5 charges, allowing a maximum injection time of 110 ms. Product ions were detected in the Orbitrap at a resolution of 35,000. Precursors were dynamically excluded for 15.0 s.

UNC-54/MHC B co-immunoprecipitation mass spectrometry analysis

Worms were grown to adulthood on RNAi plates at 15°C and transferred to 25°C for 24 h prior to sample collection (four conditions). Worms were collected and washed, and worm pellets were snap frozen and stored at –80°C until further processing. Protein from all five independent biological replicates was isolated together by sonication in NP40 lysis buffer (50 mM Tris pH 7.5, 150 mM NaCl, 1 mM EDTA, 1% NP40, 0.25% sodium deoxycholate) supplemented with

25 mM N-ethylmaleimide, 20 µM MG132, and cOmplete protease inhibitor cocktail (Roche). Protein concentration in cleared lysates was measured using a Pierce BCA protein assay kit (Thermo Fisher Scientific). 2.5 mg total protein was diluted in NP40 lysis buffer and incubated with 1 µg anti-MHC B/-UNC-54 antibody (mAb 5-8, DSHB) at 4°C overnight, while four lysate samples from *unc-54(e1301)* worms on control RNAi were incubated without antibody addition. The next day, magnetic Dynabeads Protein A (Life Technologies) were added and incubated for 1 h at 4°C. The beads were then washed six times in RIPA buffer (50 mM Tris pH 8.0, 150 mM NaCl, 1% NP40, 0.1% SDS, 0.5% Na-deoxycholate) and once in PBS, and on-bead Lys-C/trypsin digestion was performed in 8 M urea/50 mM TEAB buffer with cOmplete protease inhibitor cocktail (Roche) as for whole worm mass spectrometry, followed by mass spectrometric analysis. All 24 samples were analysed by the CECAD proteomics facility on a Q Exactive Plus Orbitrap mass spectrometer that was coupled to an EASY nLC (both Thermo Scientific). Peptides were loaded onto an in-house packed analytical column (30 cm length, 75 µm inner diameter, filled with 2.7 µm Poroshell EC120 C18, Agilent) with solvent A (0.1% formic acid in water). Peptides were chromatographically separated at a constant flow rate of 250 nL/min using the following gradient: initial 3% solvent B (0.1% formic acid in 80 % acetonitrile), 3–5% B within 1.0 min, 5–30% solvent B within 40.0 min, 30–50% solvent B within 8.0 min, 50–95% solvent B within 1.0 min, followed by washing and column equilibration. The mass spectrometer was operated in data-dependent acquisition mode. The MS1 survey scan was acquired from 300–1750 m/z at a resolution of 70,000 and a maximum injection time of 20 ms. The top 10 most abundant peptides were isolated within a 1.8 Th window and subjected to HCD fragmentation at a normalized collision energy of 27%. The AGC target was set to 5e5 charges, allowing a maximum injection time of 110 ms. Product ions were detected in the Orbitrap at a resolution of 35,000. Precursors were dynamically excluded for 10.0 s.

MaxQuant search settings and data analysis

All raw mass spectrometry data were processed with Maxquant (version 2.0.3.0)[5] using default parameters against the *C. elegans* WormBase database (PRJNA13758, version WS277, downloaded 05.08.2020). For IP samples, a chimeric database was created containing both the WormBase proteins and any generated constructs. The match-between-runs option was enabled between replicates. Follow-up analysis was performed in Perseus 1.6.15[2]. Protein groups were filtered for potential contaminants and insecure identifications. Remaining IDs were filtered for data completeness in at least one group, missing values were imputed by sigma downshift (0.3 σ width, 1.8 σ downshift), and FDR-controlled two-tailed Student's t-tests were performed. Finally, WormBase transcript IDs were converted to Uniprot IDs from Swissprot and TrEMBL data using the Uniprot ID mapping webtool. The resulting Uniprot IDs were matched to the dataset using the "matching rows by name" function, and Uniprot IDs or WormBase Gene IDs were used for data annotation and finally enrichment analyses.

Supplementary References

1. Love, M. I., Huber, W. & Anders, S. Moderated estimation of fold change and dispersion for RNA-seq data with DESeq2. *Genome Biol.* **15**, 550 (2014).
2. Tyanova, S. *et al.* The Perseus computational platform for comprehensive analysis of (prote)omics data. *Nat. Methods* **13**, 731–740 (2016).
3. Nolte, H., MacVicar, T. D., Tellkamp, F. & Krüger, M. Instant Clue: A Software Suite for Interactive Data Visualization and Analysis. *Sci. Rep.* **8**, 12648 (2018).
4. Ewels, P. A. *et al.* The nf-core framework for community-curated bioinformatics pipelines. *Nat. Biotechnol.* **38**, 276–278 (2020).
5. Tyanova, S., Temu, T. & Cox, J. The MaxQuant computational platform for mass spectrometry-based shotgun proteomics. *Nat. Protoc.* **11**, 2301–2319 (2016).
