# Supplementary material for: Optogenetic induction of mechanical muscle stress identifies myosin regulatory ubiquitin ligase NHL-1 in C. elegans
Source: Nat Commun. 2024 Aug 11;15:6879. doi: 10.1038/s41467-024-51069-3 (PMC11317515; doi:10.1038/s41467-024-51069-3)
Supplement: Supplementary file 3 — Description of Additional Supplementary Files [file 41467_2024_51069_MOESM3_ESM.pdf]

Description of Additional Supplementary Files

Supplementary Movie 1. OptIMMuS triggers sustained muscle contraction in *C. elegans*. a, Video of transgenic worms expressing the ChR2(C128S;H134R) in body wall muscle cells supplemented with ATR shows contraction, paralysis, and expulsion of eggs when the worms are exposed to a single 5-s blue light pulse.

Supplementary Movie 2. Blue light exposure still triggers muscle contraction after 3 h of OptIMMuS treatment. a, Video of transgenic worms expressing the ChR2(C128S;H134R) in body wall muscle cells supplemented with ATR shows contraction and slow movement when the worms are exposed to a 5-s blue light pulse after having been treated in the OptIMMuS incubator with 10 5-s blue light pulses (total treatment time 3 h).

Supplementary Data 1. List of proteins that are significantly changed in transcript after OptIMMuS. Proteomic log2(Fold Change) was computed from LFQlog2 in OptIMMuS versus control without ATR and the p-values for protein differences were derived from a two-tailed Student's t-test without adjustments. Transcriptomic log2(Fold Change) values and p-values were generated using DESeq2 with an apeglm shrinkage applied to the log2FC values without adjustments.

Supplementary Data 2. List of proteins that are significantly changed in protein after OptIMMuS. Proteomic log2(Fold Change) was computed from LFQlog2 in OptIMMuS versus control without ATR and the p-values for protein differences were derived from a two-tailed Student's t-test without adjustments. Transcriptomic log2(Fold Change) values and p-values were generated using DESeq2 with an apeglm shrinkage applied to the log2FC values without adjustments.

Supplementary Data 3. Lists of GO terms found in over-representation analysis of protein-transcript quadrants after OptIMMuS. Functional enrichment analysis for GO terms of Biological Processes, Cellular Component, and Molecular Function was performed on the candidates in each quadrant using the clusterProfiler package by bioconductor. Over-representation analysis was

performed using the hypergeometric test with a p-value cut-off of 0.05. Results for each quadrant are given on separate sheets labelled from 3a to 3h.

Supplementary Data 4. Lists of proteins identified in the UNC-45 proximity experiments under non-stress conditions and under OptIMMuS-induced mechanical stress conditions. Results of the statistical analysis performed in Perseus 1.6.15 are given. Protein groups were filtered for potential contaminants and insecure identifications. Remaining IDs were filtered for data completeness in at least one group, missing values were imputed by sigma downshift (0.3 σ width, 1.8 σ downshift), and one-way ANOVA and FDR-controlled two-tailed Student’s t-tests were performed. Results for the non-stress experiment and the OptIMMuS experiment are given on separate sheets labelled 4a and 4b, respectively.

Supplementary Data 5. List of proteins identified in the UNC-54(G387R)/myosin co-immunoprecipitation mass spectrometry experiment. Results of the statistical analysis performed in Perseus 1.6.15 are given. Protein groups were filtered for potential contaminants and insecure identifications. Remaining IDs were filtered for data completeness in at least one group, missing values were imputed by sigma downshift (0.3 σ width, 1.8 σ downshift), and one-way ANOVA and FDR-controlled two-tailed Student’s t-tests were performed.

Supplementary Data 6. Gene set enrichment analysis of UNC-54/MHCB/myosin interacting proteins in *nhl-1* knockdown compared to control knockdown. Ranked protein lists generated from the statistical analysis in Perseus 1.6.15 and a two-sided Student’s t-test of UNC-54/ MHC B co-immunoprecipitation and mass spectrometry were subjected to gene set enrichment analysis (GSEA) using GSEA 4.1.0 with default settings for preranked GSEA. Results for a positive or negative enrichment are given on separate sheets labelled 6a and 6b, respectively.

Supplementary Data 7. *C. elegans* and bacterial strains used in this study.

Supplementary Data 8. Oligonucleotides used in this study.

Supplementary Data 9. Antibodies used in this study.
