# Supplementary material for: Optogenetic induction of mechanical muscle stress identifies myosin regulatory ubiquitin ligase NHL-1 in C. elegans
Source: Nat Commun. 2024 Aug 11;15:6879. doi: 10.1038/s41467-024-51069-3 (PMC11317515; doi:10.1038/s41467-024-51069-3)
Supplement: Supplementary file 15 — Reporting Summary [file 41467_2024_51069_MOESM15_ESM.pdf]

nature portfolio

Corresponding author(s): Thorsten Hoppe

Last updated by author(s): Jul 17, 2024

Reporting Summary

Nature Portfolio wishes to improve the reproducibility of the work that we publish. This form provides structure for consistency and transparency in reporting. For further information on Nature Portfolio policies, see our Editorial Policies and the Editorial Policy Checklist.

Statistics

For all statistical analyses, confirm that the following items are present in the figure legend, table legend, main text, or Methods section.

n/a	Confirmed	
☐	☒	The exact sample size (*n*) for each experimental group/condition, given as a discrete number and unit of measurement
☐	☒	A statement on whether measurements were taken from distinct samples or whether the same sample was measured repeatedly
☐	☒	The statistical test(s) used AND whether they are one- or two-sided<br>*Only common tests should be described solely by name; describe more complex techniques in the Methods section.*
☒	☐	A description of all covariates tested
☐	☒	A description of any assumptions or corrections, such as tests of normality and adjustment for multiple comparisons
☐	☒	A full description of the statistical parameters including central tendency (e.g. means) or other basic estimates (e.g. regression coefficient) AND variation (e.g. standard deviation) or associated estimates of uncertainty (e.g. confidence intervals)
☐	☒	For null hypothesis testing, the test statistic (e.g. *F*, *t*, *r*) with confidence intervals, effect sizes, degrees of freedom and *P* value noted<br>*Give P values as exact values whenever suitable.*
☒	☐	For Bayesian analysis, information on the choice of priors and Markov chain Monte Carlo settings
☒	☐	For hierarchical and complex designs, identification of the appropriate level for tests and full reporting of outcomes
☒	☐	Estimates of effect sizes (e.g. Cohen's *d*, Pearson's *r*), indicating how they were calculated

Our web collection on statistics for biologists contains articles on many of the points above.

Software and code

Policy information about availability of computer code

Data collection	Zen 2.6 Pro, ARENA WMicrotracker Software (NemaMetrix), Image Studio 5.2, ImageLab 6.0.1, BioRad CFX Manager Software 3.1, WormLab Software
Data analysis	ImageJ 1.53, Zen 2.6 Pro, R version 4.0.3, RNA-seq pipeline (v3.0) from nf-core, DESeq2 (v1.30.1), apeglm (v1.12.0), clusterProfiler (v3.18.1), MaxQuant 2.0.3, Perseus 1.6.15, InstantClue, GSEA 4.1.0, REVIGO.irb.hr, BioRad CFX Manager Software 3.1, Graphpad Prism 7

For manuscripts utilizing custom algorithms or software that are central to the research but not yet described in published literature, software must be made available to editors and reviewers. We strongly encourage code deposition in a community repository (e.g. GitHub). See the Nature Portfolio guidelines for submitting code & software for further information.

Data

Policy information about availability of data

All manuscripts must include a data availability statement. This statement should provide the following information, where applicable:

- Accession codes, unique identifiers, or web links for publicly available datasets
- A description of any restrictions on data availability
- For clinical datasets or third party data, please ensure that the statement adheres to our policy

The authors declare that the main data supporting the findings of this study are available within the article and its Supplementary Informaton files. Source data are provided with the paper. Extra data are available from the corresponding author upon request.

RNA-seq data that support the findings of this study have been deposited in the Gene Expression Omnibus (GEO) repository under accession code GSE241906 [https://www.ncbi.nlm.nih.gov/geo/query/acc.cgi?acc=GSE241906]. Mass spectrometry data that support the findings of this study have been deposited in ProteomeXchange under accession codes PXD045078 [http://www.ebi.ac.uk/pride/archive/projects/PXD045078] (whole worm proteomics), PXD045081 [http://www.ebi.ac.uk/pride/archive/projects/PXD045081] (proximity proteomics), and PXD045076 [http://www.ebi.ac.uk/pride/archive/projects/PXD045076] (UNC-54/myosin Co-IP). Plasmids and C. elegans strains generated in this study will be distributed to other researchers upon request.

Research involving human participants, their data, or biological material

Policy information about studies with human participants or human data. See also policy information about sex, gender (identity/presentation), and sexual orientation and race, ethnicity and racism.

Reporting on sex and gender	*Use the terms sex (biological attribute) and gender (shaped by social and cultural circumstances) carefully in order to avoid confusing both terms. Indicate if findings apply to only one sex or gender; describe whether sex and gender were considered in study design; whether sex and/or gender was determined based on self-reporting or assigned and methods used.* *Provide in the source data disaggregated sex and gender data, where this information has been collected, and if consent has been obtained for sharing of individual-level data; provide overall numbers in this Reporting Summary. Please state if this information has not been collected.* *Report sex- and gender-based analyses where performed, justify reasons for lack of sex- and gender-based analysis.*
Reporting on race, ethnicity, or other socially relevant groupings	*Please specify the socially constructed or socially relevant categorization variable(s) used in your manuscript and explain why they were used. Please note that such variables should not be used as proxies for other socially constructed/relevant variables (for example, race or ethnicity should not be used as a proxy for socioeconomic status).* *Provide clear definitions of the relevant terms used, how they were provided (by the participants/respondents, the researchers, or third parties), and the method(s) used to classify people into the different categories (e.g. self-report, census or administrative data, social media data, etc.)* *Please provide details about how you controlled for confounding variables in your analyses.*
Population characteristics	*Describe the covariate-relevant population characteristics of the human research participants (e.g. age, genotypic information, past and current diagnosis and treatment categories). If you filled out the behavioural & social sciences study design questions and have nothing to add here, write "See above."*
Recruitment	*Describe how participants were recruited. Outline any potential self-selection bias or other biases that may be present and how these are likely to impact results.*
Ethics oversight	*Identify the organization(s) that approved the study protocol.*

Note that full information on the approval of the study protocol must also be provided in the manuscript.

Field-specific reporting

Please select the one below that is the best fit for your research. If you are not sure, read the appropriate sections before making your selection.

☒ Life sciences ☐ Behavioural & social sciences ☐ Ecological, evolutionary & environmental sciences

For a reference copy of the document with all sections, see nature.com/documents/nr-reporting-summary-flat.pdf

Life sciences study design

All studies must disclose on these points even when the disclosure is negative.

Sample size	Sample size determination was done according to standard C. elegans approaches. Exact sample sizes are stated in the respective figure legends, methods section, and Supplementary Information. No statistical method was used to determine dample size. Overall at least three biological replicates were performed for each experiment, which is an established norm in the scientific community.
Data exclusions	No data were excluded from analyses.
Replication	At least three biological replicates with similar outcome were performed for each experiment, except for TEM in Figure 2a,b, S2 (n=1), fluorescence microscopy S4e (n=1) and 4a, S5a (n=2) and Western blots S6a (n =1) and S6d (n=2) as described in the figure legends.
Randomization	For the experimental setups in this study randomization was not applicable.
Blinding	For the experimental setups in this study blinding was not applicable.

Reporting for specific materials, systems and methods

We require information from authors about some types of materials, experimental systems and methods used in many studies. Here, indicate whether each material, system or method listed is relevant to your study. If you are not sure if a list item applies to your research, read the appropriate section before selecting a response.

Materials & experimental systems

n/a	Involved in the study	
☐	☒	Antibodies
☒	☐	Eukaryotic cell lines
☒	☐	Palaeontology and archaeology
☐	☒	Animals and other organisms
☒	☐	Clinical data
☒	☐	Dual use research of concern
☒	☐	Plants

Methods

n/a	Involved in the study	
☒	☐	ChIP-seq
☒	☐	Flow cytometry
☒	☐	MRI-based neuroimaging

Antibodies

Antibodies used

Mouse monoclonal anti alpha tubulin (clone B-5-1-2), Sigma Aldrich, Cat# T6074, RRID:AB_477582;
Mouse monoclonal anti-FLAG (M2), Sigma Aldrich, Cat# F3162, RRID:AB_259529;
Rabbit polyclonal anti-HA, Sigma Aldrich, Cat# H6908, RRID:AB_260070;
Rabbit polyclonal anti-UNC-45, Hoppe Lab/Biogenes Berlin N/A - custom antibody;
Mouse monoclonal anti-UNC-54/MHC-B (mAb 5-8), Developmental Studies Hybridoma Bank, Cat# mAb 5-8, RRID:N/A;
IRDye® 800CW Donkey anti-Mouse IgG (H + L), LI-COR Biosciences, Cat# 926-32212, RRID:AB_2716622;
IRDye® 800CW Donkey anti-Rabbit IgG (H + L), LI-COR Biosciences, Cat# 926-32213, RRID:AB_621848;
IRDye® 680 Donkey anti-Mouse IgG (H + L), LI-COR Biosciences, Cat# 962-68072, RRID:AB_621844;
IRDye® 680 Donkey anti-Rabbit IgG (H + L), LI-COR Biosciences, Cat# 962-68073, RRID:AB_621845;
IRDye® 800CW Streptavidin, LI-COR Biosciences, Cat# 926-32230
Goat anti-Rabbit IgG (H+L) Cross-Adsorbed Secondary Antibody, Alexa Fluor™ 488 Invitrogen Cat# A-11008 RRID:AB_143165

Validation

Validations of primary and secondary antibodies were done by the stated manufacturer's or by the laboratory they where produced by.

Mouse monoclonal anti-alpha-tubulin (clone B-5-1-2), Mouse monoclonal anti-FLAG (M2), Rabbit polyclonal anti-HA:
Independent Antibody Verification – Demonstrating antibody specificity through the use of multiple antibodies against target in IHC or ICC. (https://www.sigmaaldrich.com/DE/de/technical-documents/technical-article/protein-biology/immunohistochemistry/antibody-enhanced-validation)

Mouse monoclonal anti-UNC-54/MHC-B (mAb 5-8):
No rating or validation information has been found for Mouse monoclonal anti UNC-54/MHC B. mAb 5-8 was deposited to the DSHB by Miller, David, M., III (DSHB Hybridoma Product mAb 5-8).

Animals and other research organisms

Policy information about studies involving animals; ARRIVE guidelines recommended for reporting animal research, and Sex and Gender in Research

Laboratory animals

The study involved Caenorhabditis elegans strains of various genotypes listed in the provided Supplementary Information. C. elegans populations entail predominantly hermaphrodites. The tested animals were all young adult to adult day 1 stage.

Wild animals

The study did not involve wild animals.

Reporting on sex

All experiments in this study were performed on hermaphrodite C. elegans populations.

Field-collected samples

The study did not involve samples collected from the field.

Ethics oversight

This study only includes work with the nematode C. elegans. No ethical approval or guidance is required.

Note that full information on the approval of the study protocol must also be provided in the manuscript.

Plants

Seed stocks	*Report on the source of all seed stocks or other plant material used. If applicable, state the seed stock centre and catalogue number. If plant specimens were collected from the field, describe the collection location, date and sampling procedures.*
Novel plant genotypes	*Describe the methods by which all novel plant genotypes were produced. This includes those generated by transgenic approaches, gene editing, chemical/radiation-based mutagenesis and hybridization. For transgenic lines, describe the transformation method, the number of independent lines analyzed and the generation upon which experiments were performed. For gene-edited lines, describe the editor used, the endogenous sequence targeted for editing, the targeting guide RNA sequence (if applicable) and how the editor was applied.*
Authentication	*Describe any authentication procedures for each seed stock used or novel genotype generated. Describe any experiments used to assess the effect of a mutation and, where applicable, how potential secondary effects (e.g. second site T-DNA insertions, mosiacism, off-target gene editing) were examined.*
