# Supplementary figures and images for: Optogenetic induction of mechanical muscle stress identifies myosin regulatory ubiquitin ligase NHL-1 in C. elegans
Source: Nat Commun. 2024 Aug 11;15:6879. doi: 10.1038/s41467-024-51069-3 (PMC11317515; doi:10.1038/s41467-024-51069-3)

C

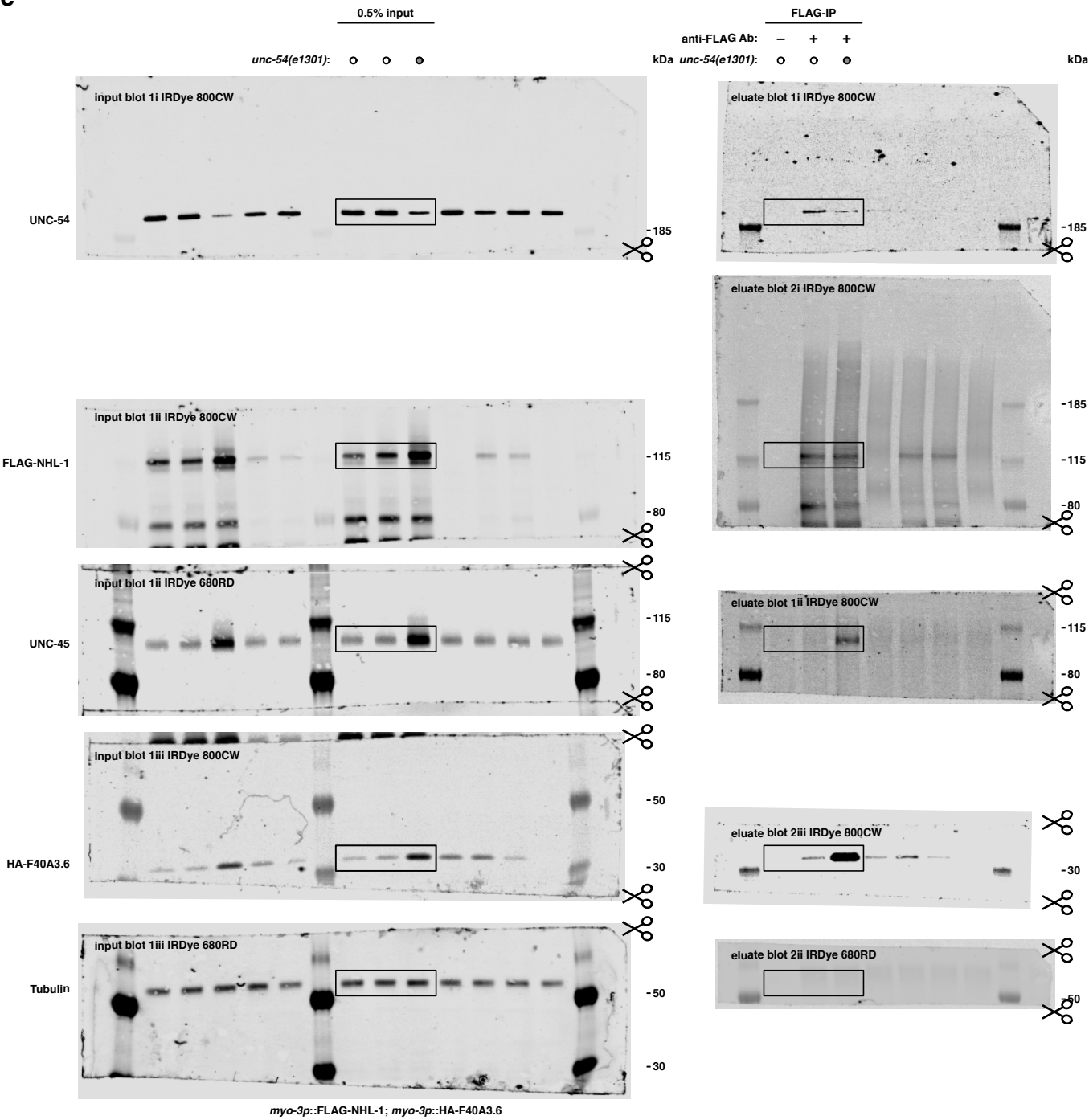

Supplement: Supplementary file 16 — Source Data [file 41467_2024_51069_MOESM16_ESM.zip › Kutzner_Source_Data/Kutzner_SourceData_Fig4_UncroppedBlots.pdf]

e

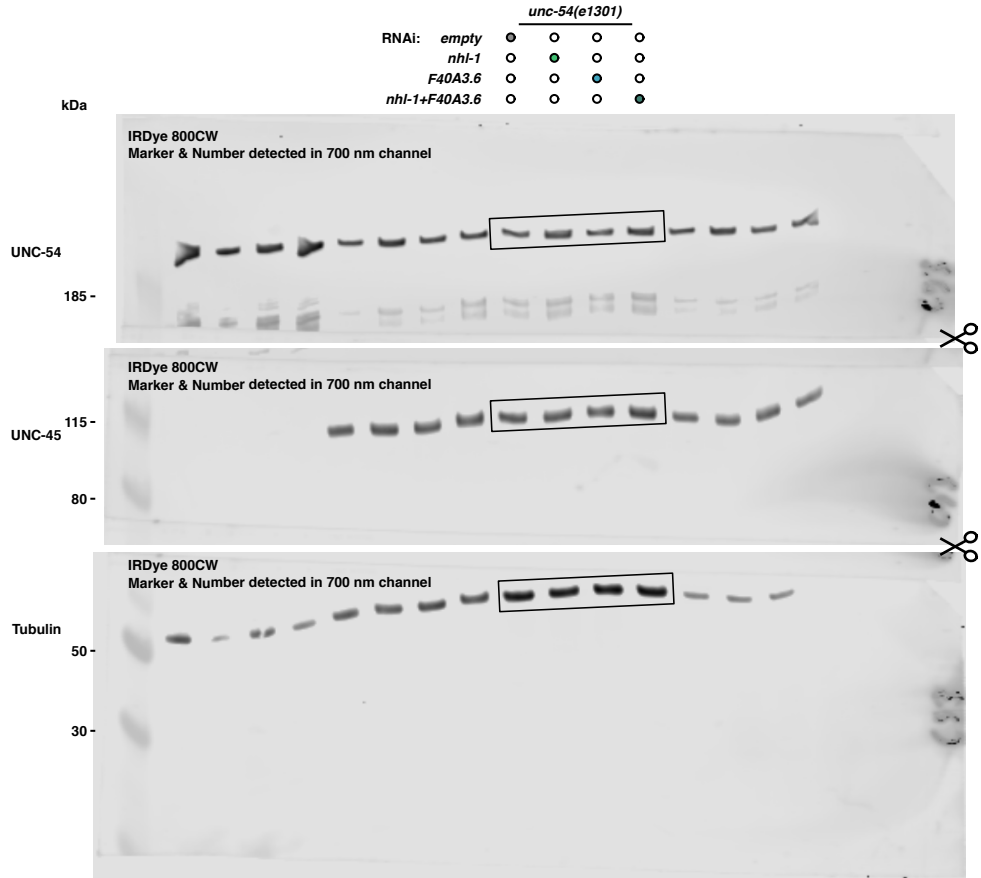

Supplement: Supplementary file 16 — Source Data [file 41467_2024_51069_MOESM16_ESM.zip › Kutzner_Source_Data/Kutzner_SourceData_Fig5_UncroppedBlots.pdf]

**b**

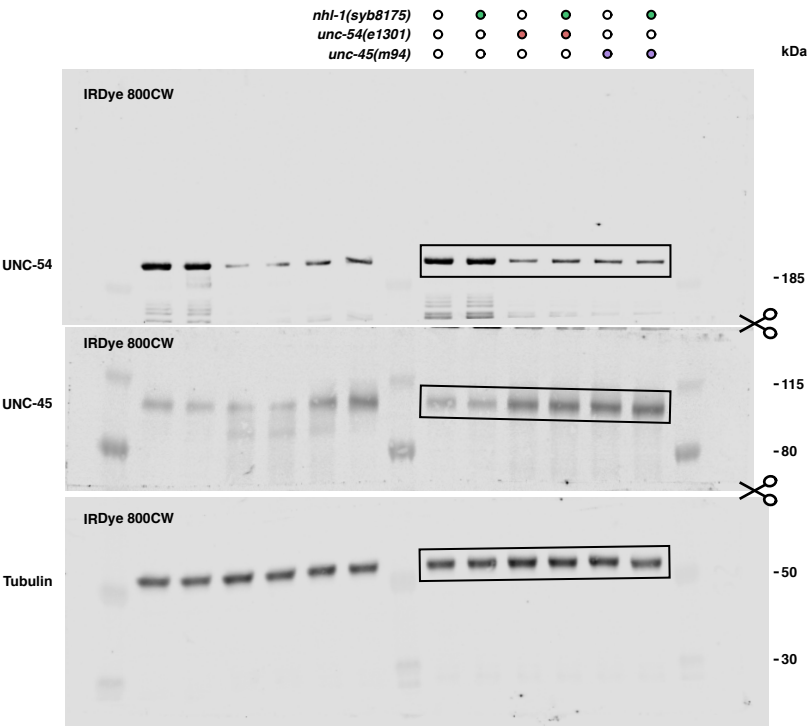

Supplement: Supplementary file 16 — Source Data [file 41467_2024_51069_MOESM16_ESM.zip › Kutzner_Source_Data/Kutzner_SourceData_Fig6_UncroppedBlots.pdf]

d

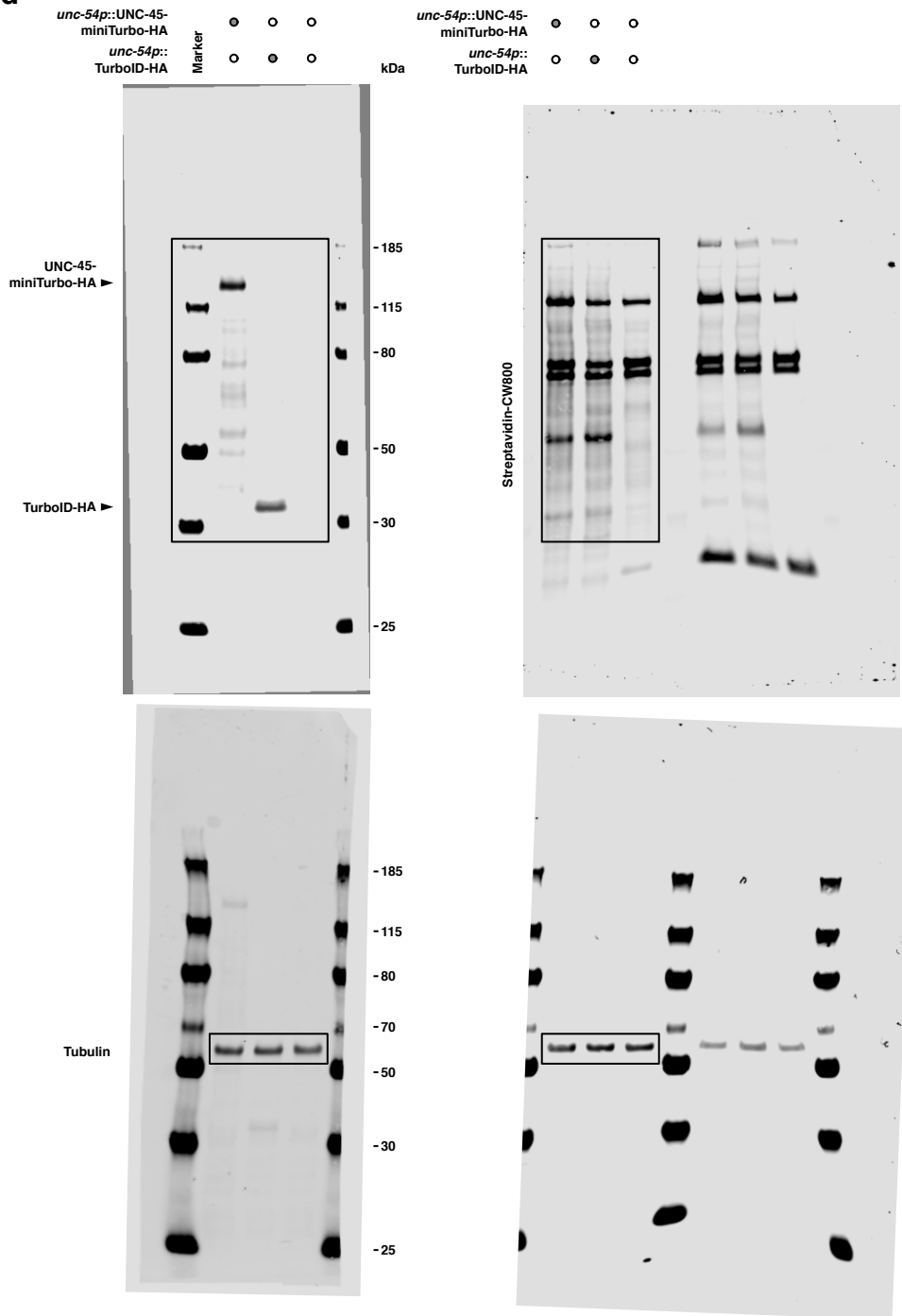

Supplement: Supplementary file 16 — Source Data [file 41467_2024_51069_MOESM16_ESM.zip › Kutzner_Source_Data/Kutzner_SourceData_SuppFigS4_UncroppedBlots.pdf]

b

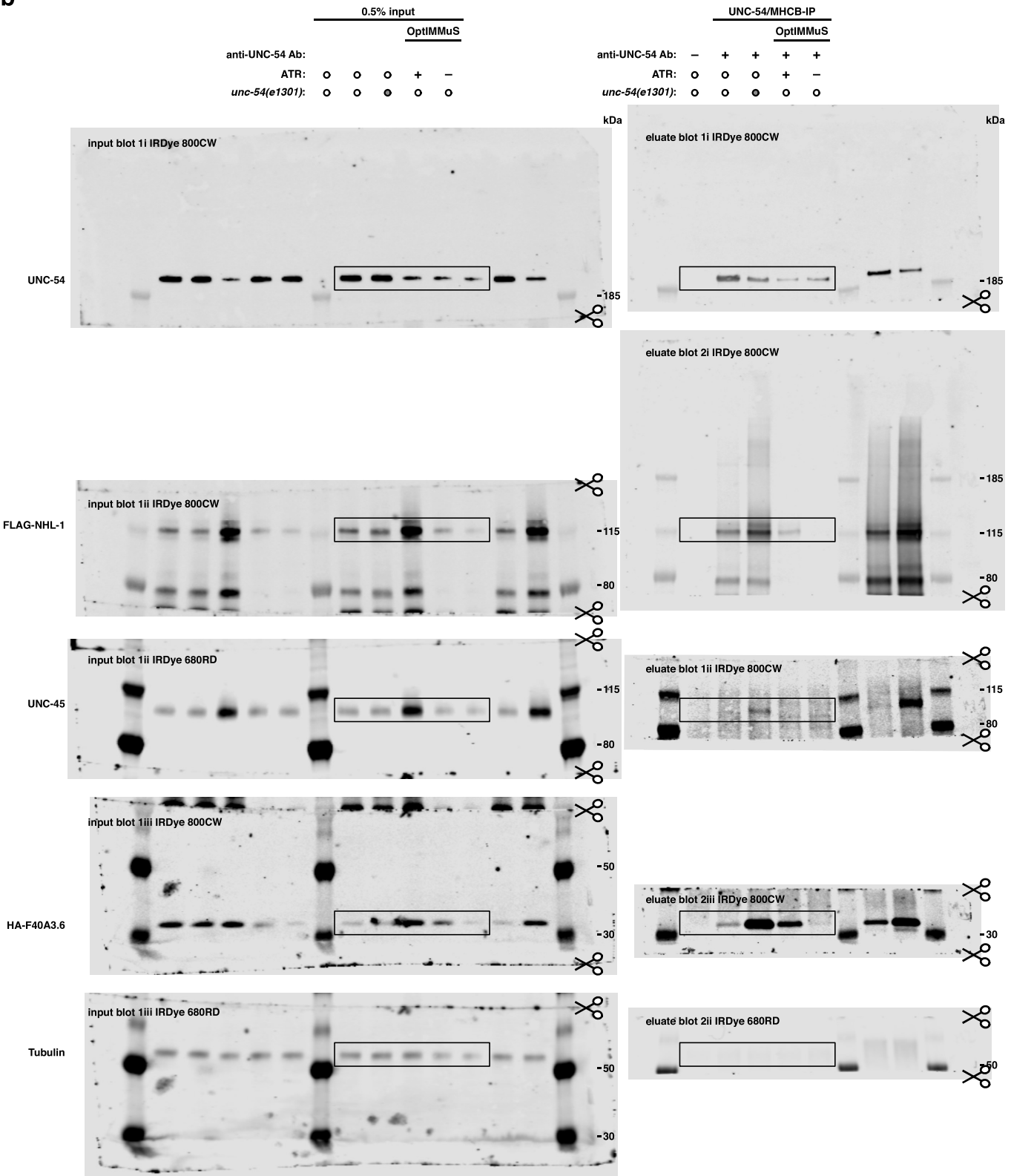

C

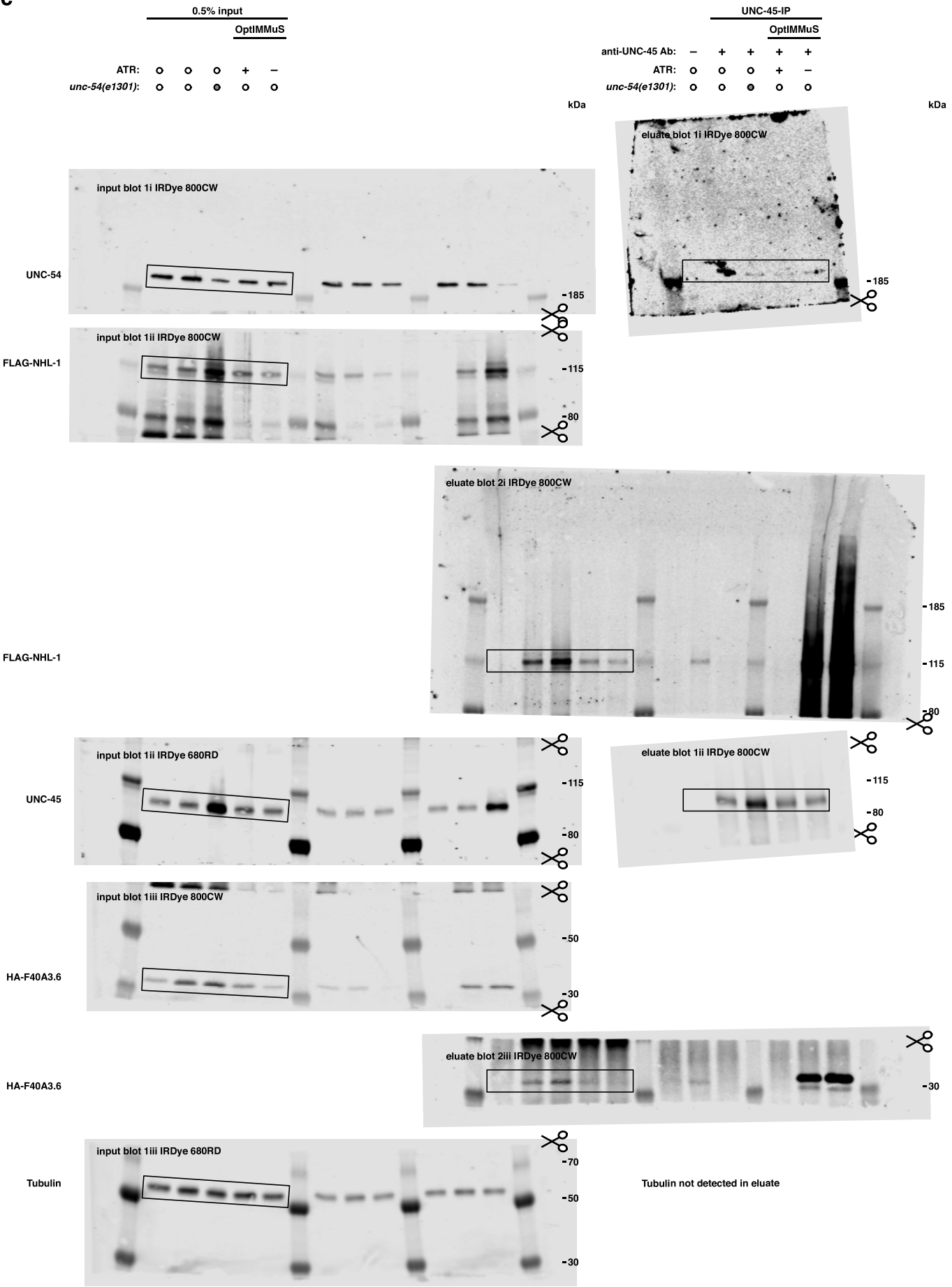

Supplement: Supplementary file 16 — Source Data [file 41467_2024_51069_MOESM16_ESM.zip › Kutzner_Source_Data/Kutzner_SourceData_SuppFigS5_UncroppedBlots.pdf]

**a**

|       |                               |   |   |   |   |
|-------|-------------------------------|---|---|---|---|
| RNAi: | control                       | ● | ○ | ○ | ○ |
|       | <i>nhl-1</i>                  | ○ | ● | ○ | ○ |
|       | <i>F40A3.6</i>                | ○ | ○ | ● | ○ |
|       | <i>nhl-1</i> + <i>F40A3.6</i> | ○ | ○ | ○ | ● |

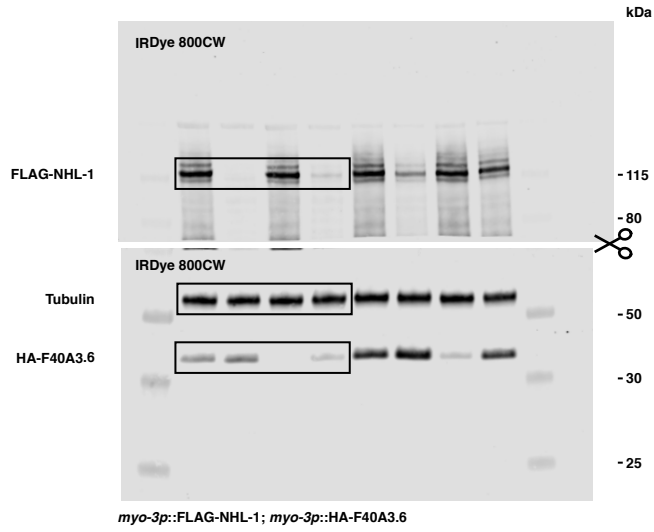

**d**

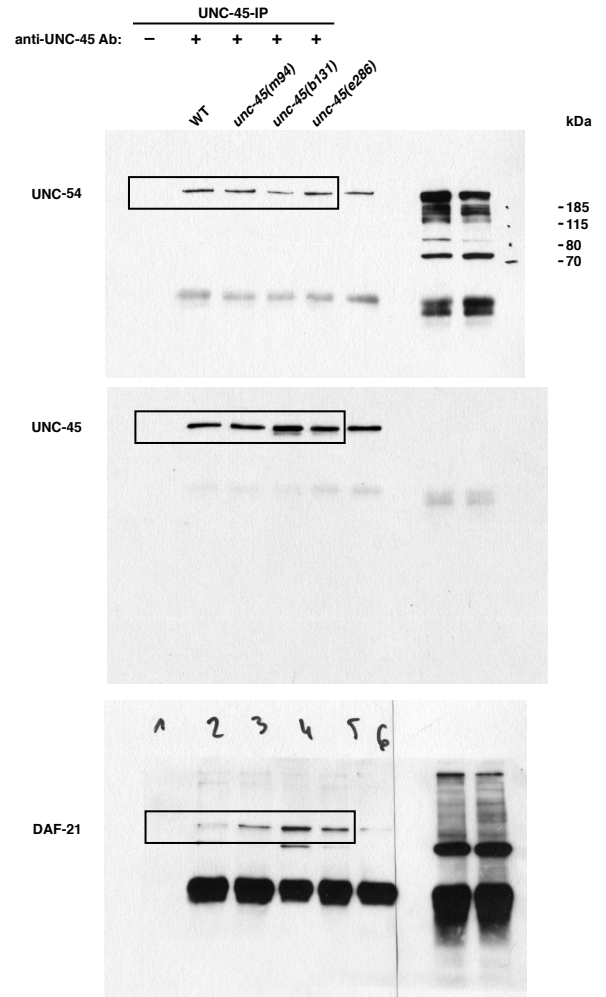

Supplement: Supplementary file 16 — Source Data [file 41467_2024_51069_MOESM16_ESM.zip › Kutzner_Source_Data/Kutzner_SourceData_SuppFigS6_UncroppedBlots.pdf]

a

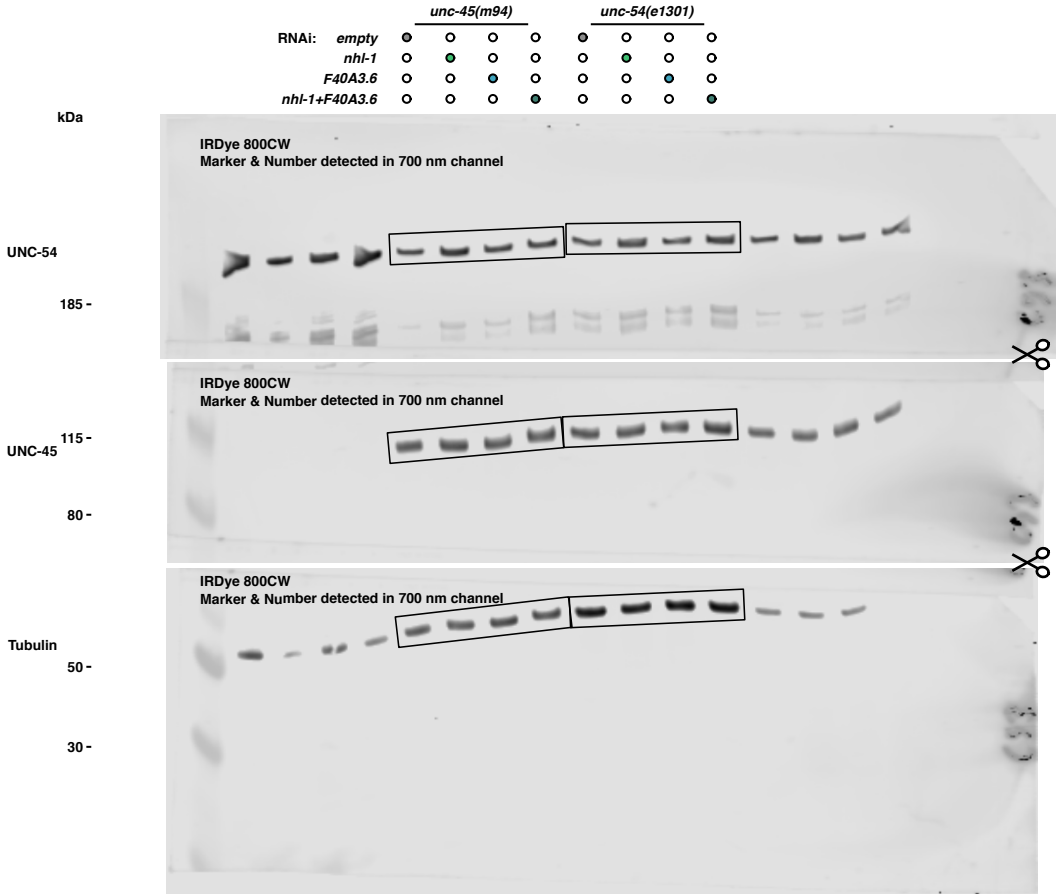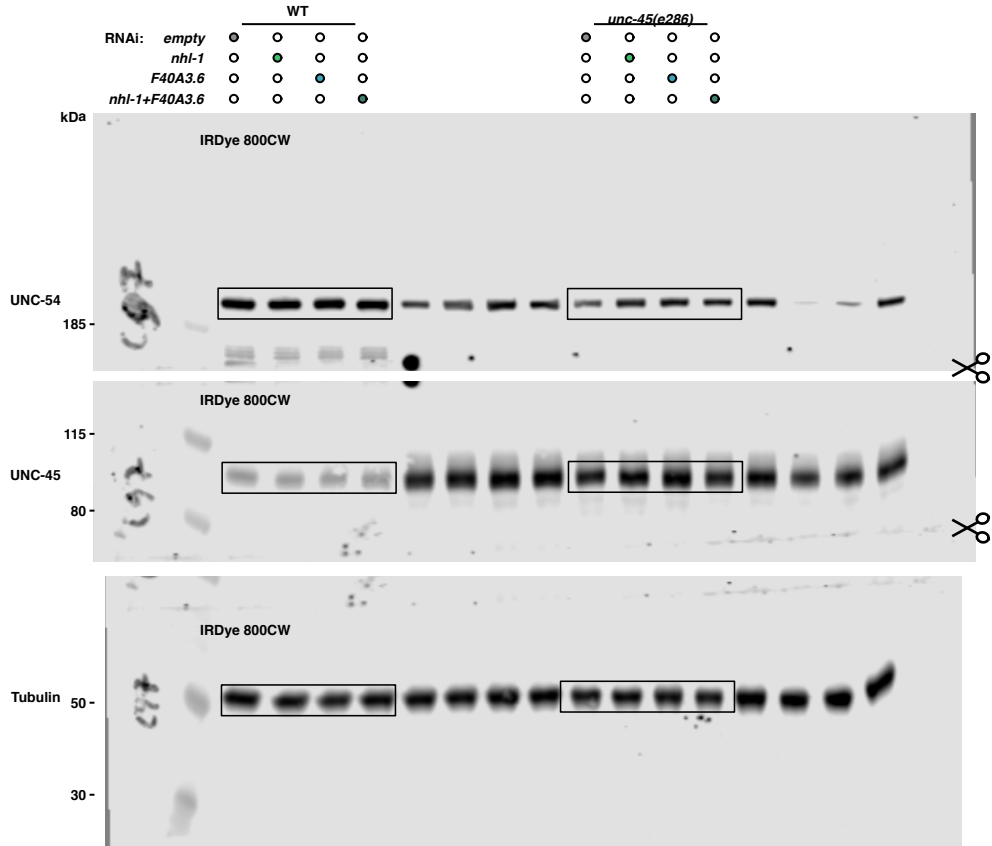

a

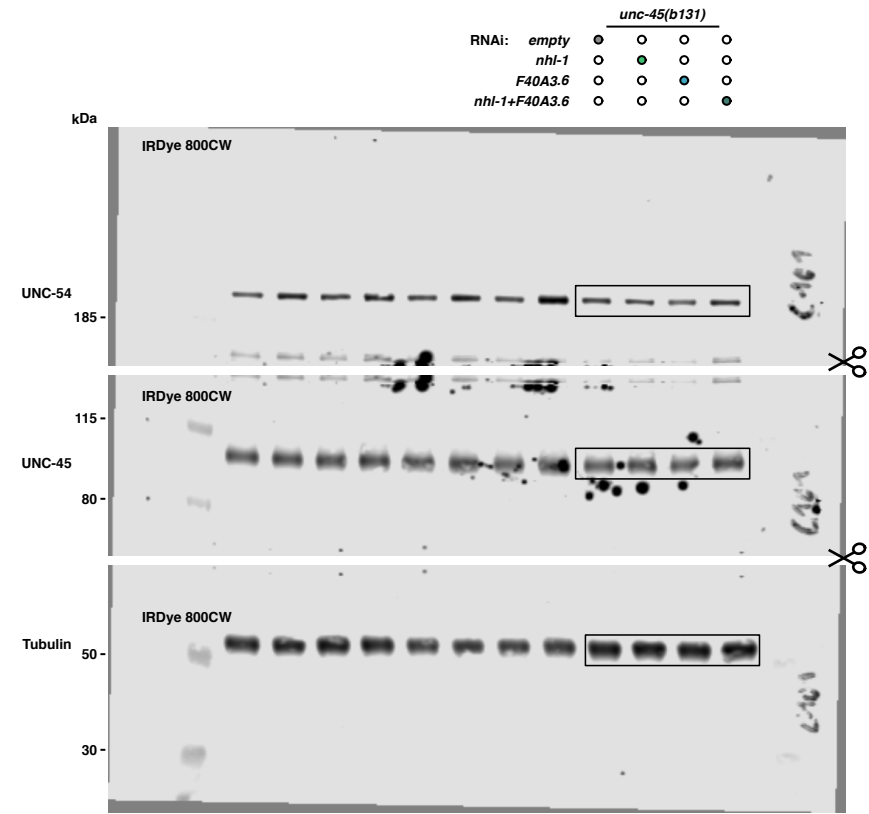

d

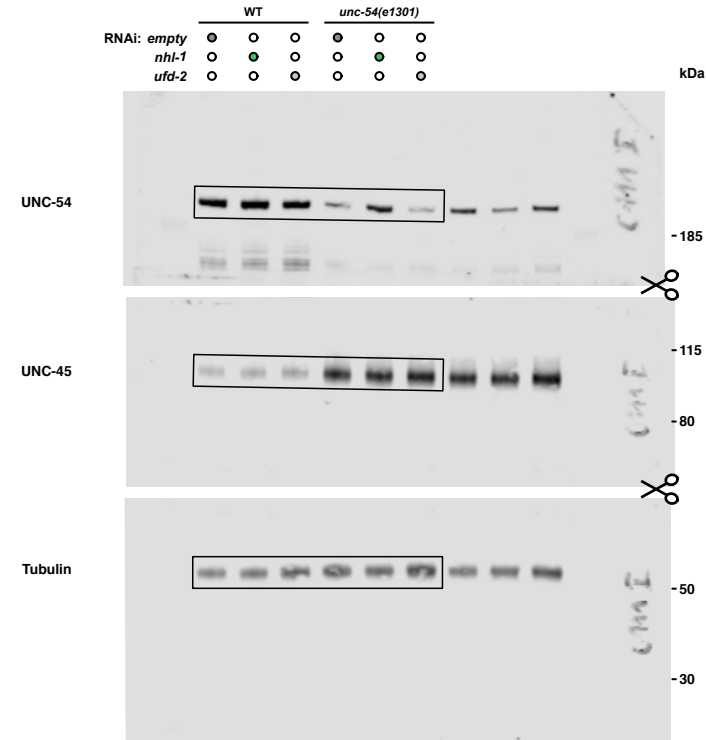

Supplement: Supplementary file 16 — Source Data [file 41467_2024_51069_MOESM16_ESM.zip › Kutzner_Source_Data/Kutzner_SourceData_SuppFigS7_UncroppedBlots.pdf]
